# Psychometric properties of the Epworth Sleepiness Scale in Brazilian medical students

**DOI:** 10.1055/s-0045-1804921

**Published:** 2025-03-19

**Authors:** Renato Canevari Dutra da Silva, Anderson Garcez, Adriana Vieira Macedo Brugnoli, Marcos Pascoal Pattussi, Maria Teresa Anselmo Olinto

**Affiliations:** 1 Universidade do Vale do Rio dos Sinos, Programa de Pós-Graduação em Saúde Coletiva, São Leopoldo RS, Brazil.; 2 Universidade do Rio Verde, Rio Verde GO, Brazil.; 3 Universidade Federal do Rio Grande do Sul, Programa de Pós-Graduação em Ciências Médicas: Endocrinologia, Porto Alegre RS, Brazil.; 4 Universidade Federal de Ciências da Saúde de Porto Alegre, Programa de Pós-Graduação em Ciências da Nutrição, Porto Alegre RS, Brazil.; 5 Universidade Federal do Rio Grande do Sul, Programa de Pós-Graduação em Alimentação, Nutrição e Saúde, Porto Alegre RS, Brazil.

**Keywords:** Disorders of Excessive Somnolence, Sleepiness, Epworth Sleepiness Scale, Students, Medical

## Abstract

**Background** Excessive daytime sleepiness (EDS) refers to the propensity to become drowsy or fall asleep when the intention and expectation would be to stay awake, and the Epworth Sleepiness Scale (ESS) is an easy-to-apply instrument that can be used to assess the presence of EDS.

**Objective** To evaluate the psychometric properties of the ESS, including its construct validity and internal consistency, in a population of university students.

**Methods** Two samples of 400 students from the medicine program of a university located in the Midwest of Brazil were randomly selected from a cross-sectional academic study conducted in 2018. Construct validity was examined through exploratory and confirmatory factor analysis of the eight items of the ESS, and the internal consistency was evaluated using the Cronbach's α coefficient (α).

**Results** It was found that factor analyses revealed better adjustment measures when considering the ESS to be two-dimensional, grouped into two main factors: the first factor referring to the evaluation of sleepiness at rest, and the second referring to drowsiness in activity (standardized root mean square residual [SRMR] = 0.053; root mean square error of approximation [RMSEA] = 0.095; Comparative Fit Index [CFI] = 0.937; and Tucker-Lewis Index [TLI] = 0.908; *p* < 0.05). Moreover, the ESS presented an adequate internal consistency (α = 0.75).

**Conclusion** The present study showed general psychometric properties adequate for the ESS in medical students, including an acceptable construct validity and internal consistency. Thus, the ESS may be suitable to assess EDS in university students, especially medical students.

## INTRODUCTION

Excessive daytime sleepiness (EDS) has been described as a symptom that arises at any time and refers to the propensity to get drowsy or even fall asleep when the intention and expectation would be to stay awake. 1 It is associated with difficulty in maintaining attention and impairment in reasoning and memory in addition to affecting the quality of life and academic and professional performance. 2

Several methods and tools have been developed to assess EDS, ranging from detailed clinical investigation to specific tests such as polysomnography, wakefulness maintenance test, and multiple sleep latency testing. 3 There are also subjective tests, such as scales that evaluate EDS through the individual's self-report, among which the Epworth Sleepiness Scale (ESS) can be cited. 1

The ESS was originally developed in English to evaluate EDS in adult populations; however, it has also been successfully used among children and adolescents. 4 It has been translated into several languages and used in different cultures, 5 6 7 including the Brazilian population. 8 It has been used both in populations in general 9 as well as in clinical evaluations. 10

The ESS, in its original validation study, presented a high level of internal consistency, with Cronbach's α ranging from 0.88 for individuals with sleep disorders to 0.73 for medical university students. 11 In the only ESS validation study for the Brazilian population, the scale was tested in patients aged 18 to 65 years with specific characteristics, including evaluation for primary snoring, insomnia, and obstructive sleep apnea-hypopnea syndrome compared with a control group with normal sleep habits and without apparent snoring, which presented an internal consistency with a Cronbach's α of 0.83. 8 However, in addition to the population being specific, only the first validation stage was performed: translation and back translation.

Construct validity seeks to test whether the instrument can adequately represent the theoretical construct that it seeks to measure; thus, one can determine whether the ESS evaluates EDS in a valid and precise way, according to the principles of psychometrics. 12 While internal consistency refers to the homogeneity of the scale items, 12 13 this can indicate whether all ESS items necessarily measure the same characteristic (EDS).

Taking into account that EDS has a high prevalence among university students, 14 15 16 and given the impact that it can have on the performance and academic training of this population, 2 the present study aimed to evaluate the psychometric properties of the ESS, especially its construct validity and internal consistency in Brazilian medical students.

## METHODS

### Samples and participants

Two samples were obtained from a cross-sectional academic study conducted in 2018, including a population of 2,295 health students (1,582 medical students) of the Rio Verde, Aparecida de Goiânia, and Goianésia campuses of Universidade do Rio Verde –(UniRV), a public university located in the Brazilian Midwest. The present study included all university students aged over 18 years enrolled in health courses across the three campuses of both sexes. Students who reported the use of medications for sleep, such as sedatives, tranquilizers, or anxiolytics, as well as pregnant or breastfeeding students, were excluded, as these conditions may have affected the outcomes. 16

For the present study, from a total of 1,582 medical students, 800 were selected to form 2 independent samples of 400 students each. This included a randomized sample of 400 students (200 of each sex) for Exploratory Factor Analysis (EFA) and another randomized sample of 400 students (200 of each sex) for Confirmatory Factor Analysis (CFA). This sample size of 400 students in each group was determined to obtain factor loadings of 0.30 or higher as statistically significant, 17 considering a statistical power of 80% and a confidence level of 95%.

The current study was submitted and approved by the Research Ethics Committees of Universidade do Vale do Rio dos Sinos (process no. 2.892.764) and UniRV (process no. 2.905.704). All students who agreed to participate in the study signed an Informed Consent Form before answering the questionnaires, according to the ethical aspects for research involving human beings arranged in resolution 466/2012 of the National Health Council of Brazil. All the student data were maintained with confidentiality and compliance with the Declaration of Helsinki. This study was reviewed by two Research Ethics Committees; however, data collection was conducted exclusively at UniRV.

### Instruments

Excessive daytime sleepiness was assessed with the Portuguese-language version of the ESS, 1 3 8 which includes a list of eight situations to mark the probability of napping or sleeping, and not just feeling tired, in each of them. The eight situations investigated are: “sitting and reading”; “watching TV”; “sitting inactive in a public place (e.g., a theater or a meeting)”; “as a passenger in a car for an hour without a break”; “lying down to rest in the afternoon when circumstances permit”; “sitting and talking to someone”; “sitting quietly after a lunch without alcohol” and “in a car, while stopped for a few minutes in traffic.”

In each of these situations, the student was instructed to respond on a 4-point scale, whose scores range from 0 to 3, in which 0 - “no chance of dozing,” 1 - “slight chance of dozing,” 2 - “moderate chance of dozing” and 3 - “high chance of dozing,” represent the different levels of sleepiness in each of the situations. The student was always oriented to consider their way of life taken recently and was instructed to imagine how it would affect them, in case any of these situations had not been encountered. The score was obtained by applying the ESS ranges from 0 to 24, calculated by adding the individual scores for each of the 8 items. The interpretation of the score is divided into four categories: 0 to 7 (“it is unlikely that you are abnormally sleepy”); 8 to 9 (“you have an average amount of daytime sleepiness”); 10 to 15 (“you may be excessively sleepy depending on the situation, and may want to consider seeking medical attention”); and 16 to 24 (“you are excessively sleepy and should consider seeking medical attention”). 1 11

In addition to the ESS, the students answered a standardized and precoded questionnaire containing sociodemographic variables (age, sex, skin color/race, marital status, and economic class), to characterize the sample. Economic class was determined using the Brazilian Association of Research Companies (Associação Brasileira de Empresas de Pesquisa – ABEP, in Portuguese) scale, which estimates the purchasing power of individuals and families based on household possessions and the education level of the head of the household. The classes were subsequently categorized as high (A), middle (B), and low (C/D/E). The questionnaire administration was conducted in the classroom, where the instructions were read aloud by the researcher responsible to facilitate the response, resolve doubts, and minimize loss of data from respondents. After its completion, the questionnaire was deposited in a sealed urn, and, subsequently, the codification of the same was performed.

### Data analysis

Double data entry was conducted using Epidata (Centers for Disease Control and Prevention, Atlanta, GA, USA), version 3.1, followed by a subsequent comparison to identify and rectify potential typos. Socioeconomic and demographic characteristics such as sex, age, skin color/race, marital status, and economic class were analyzed by means of absolute and relative frequencies.

Exploratory factor analyses (EFAs) and confirmatory factor analyses (CFAs) for the 8 ESS items were conducted in the Mplus program, version 8.4 (Muthén & Muthén, Los Angeles, CA, USA), 18 using the weighted least squarest mean and variance (WLSMV) adjusted estimator for categorical variable scan analysis. Loads with values ≥ 0.30 were considered acceptable; this is the minimum value required for the variable to be a useful representative of the factor. 19 For the interpretation of the results, Geomin oblique factorial rotation was used, because it allows the factors to be correlated with each other. 17

The CFA was performed to confirm the hypothetical factorial structure found in the EFA, thus identifying the validity of the ESS construct. To measure the absolute fit quality indices of the factorial models, we used the Chi-squared (χ ^2^ ), standardized root mean square residual (SRMR), and root mean square error of approximation (RMSEA) tests. For the χ ^2^ its value had to accept the null hypothesis, with *p* -value > 0.05. For the SRMR and RMSEA, values close to or below 0.08 and 0.06 respectively indicated a satisfactory adjustment of the model. Two other relative adjustment indexes used were the Tucker-Lewis Index (TLI) and the Comparative Fit Index (CFI), with values from 0.90 to 0.95 expected as indicative of acceptable adjustment. 20

The Cronbach's α coefficient (α) was used to evaluate the internal consistency of the scale. Internal consistency assesses whether the domains of the instrument in question measure the same characteristic, that is, they measure its homogeneity. The α coefficient corresponds to an index used to measure the reliability of the internal consistency type of a scale, that is, to evaluate the magnitude to which the items of an instrument are correlated. 13 Values of α ≥ 0.7 for each factor show a factorial structure with good internal consistency. 21

## RESULTS

Table 1 shows the distribution of samples according to the sociodemographic characteristics of the university students included in the EFA ( *n* = 400) and the CFA ( *n* = 400). A similar distribution of the evaluated characteristics was observed between the 2 samples. About ⅓ of the two samples was formed by university students aged between 20.1 and 22 years, 32.3% in the sample used in the EFA and 36.3% in the one for CFA. Most of the students in both samples, EFA and CFA, respectively, reported having white skin color (62%; 61%), not having a partner (88.97%; 90.73%), and belonging to economic class A (54.52%; 57.91%).

**Table 1:** Distribution of the samples according to the demographic and socioeconomic characteristics of university students in the Brazilian Midwest (EFA = 400; CFA = 400)

Variables	Categories	EFA Sample		CFA Sample	
		n	%	n	%
Sex	Female	200	50.0	200	50.0
	Male	200	50.0	200	50.0
Age (years)	18–20	99	24.81	89	22.31
	20.1–22	129	32.33	144	36.09
	22.1–24	104	26.79	95	23.81
	> 24	67	16.79	71	17.79
Skin color/race	White	248	62.0	244	61.0
	Brown/Black	142	35.5	143	35.75
	Other	10	2.5	13	3.25
Marital status	With partner	44	11.03	37	9.27
	Without partner	355	88.97	362	90.73
Economic class*	Class A (high)	211	54.52	227	57.91
	Class B (middle)	150	38.76	136	34.69
	Class C, D, E (low)	26	6.72	29	7.40

Abbreviations: CFA, confirmatory factor analysis; EFA, exploratory factor analysis.

Note: *Economic class was determined using the Brazilian Association of Research Companies (ABEP) scale, which estimates the purchasing power of individuals and families based on household possessions and the education level of the head of the household.

The EFA showed a good fit for both the models evaluated (unifactorial and bifactorial), fixing the extraction in one and two factors, the factor loadings presented values higher than 0.5 in all items and solutions evaluated. As expected, the two-factor solution presented a better adjustment (χ 2 = 0.627, SRMR = 0.029, RMSEA = 0.001, CFI = 1, and TLI = 1) ( Table 2 ). When evaluating the distribution of factor loadings, it was observed that questions 1, 2, 4, 5, and 7 presented higher factor loadings in factor 1, while questions 3, 6, and 8 had higher factor loadings in factor 2. Thus, factor 1 was named ‘sleepiness in a state of rest’ and those grouped in factor 2 displayed ‘active sleepiness’ ( Table 2 ). In the internal consistency analysis, Cronbach's α coefficients (α) were found with values higher than 0.7 in all factorial models tested ( Table 2 ).

**Table 2:** Exploratory factor analysis and internal consistency of the Epworth Sleepiness Scale in university students from the Brazilian Midwest, 2018 ( *n* = 400)

Epworth Sleepiness Scale**	Total Sample ( *n* = 400)				
Item	1 Factor		2 Factors		
How likely are you to doze off or fall asleep in the following situations? You should rate your chances of dozing off, not just feeling tired. Even if you have not done some of these things recently try to determine how they would have affected you.	RV	F1	RV	F1	F2
1) Sitting and reading	0.57	0.654*	0.56	0.548*	0.170
2) Watching TV	0.65	0.589*	0.60	0.631*	0.000
3) Sitting inactive in a public place (e.g., a theater or a meeting)	0.52	0.696*	0.47	0.244*	0.555*
4) As a passenger in a car for an hour without a break	0.76	0.489*	0.76	0.338*	0.203
5) Lying down to rest in the afternoon when circumstances permit	0.65	0.596*	0.55	0.699*	−0.054
6) Sitting and talking to someone	0.59	0.640*	0.40	−0.014	0.780*
7) Sitting quietly after a lunch without alcohol	0.52	0.691*	0.51	0.567*	0.190
8) In a car, while stopped for a few minutes in traffic	0.66	0.583*	0.57	0.062	0.619*
**Internal consistency - Cronbach's Alpha (α)**	–	0.75	–	0.70	0.75
Eigenvalue	–	3.643	–	3.643	0.992
Chi-squared (χ ^2^ ); *p* -value		40.685 (20); *p* = 0.0041		10.813 (13); *p* = 0.6265	
SRMR		0.062		0.029	
RMSEA		0.051		0.001	
CFI		0.979		1.000	
TLI		0.971		1.000	

Abbreviations: CFI, Comparative Fit Index; RMSEA, root mean square error of approximation; RV, residual variance; SRMR, standardized root mean square residual; TLI, Tucker-Lewis Index.

Notes: **Model with 1 factor: F1 = general sleepiness; model with 2 factors: F1 = sleepiness at rest; F2 = drowsiness in activity; commonalities: RV; Kaizer method: > 1/χ2 ( *p* -value > 0.05); Cronbach's α coefficient > 0.7; SRMR < 0.08; RMSEA < 0.06; CFI < 0.9; and TLI < 0.90.

**Figure 1:**
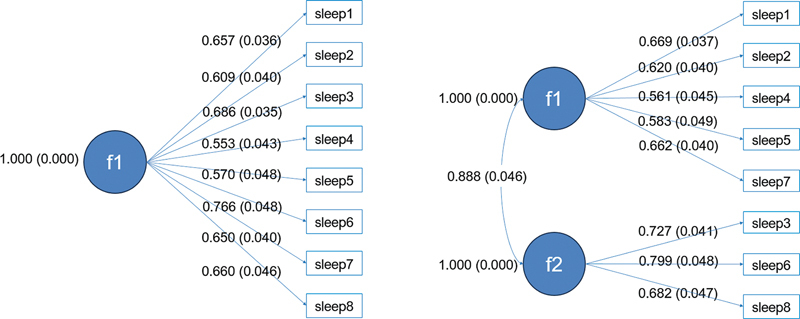
Notes: *f1: Drowsiness in General; sleep 1, sitting and reading; sleep 2, watching TV; sleep 3, sitting inactive in a public place (such as a theater or a meeting); sleep 4, as a passenger in a car for an hour without a break; sleep 5, lying down to rest in the afternoon when circumstances permit; sleep 6, sitting and talking to someone; sleep 7, sitting quietly after a lunch without alcohol; sleep 8, in a car, while stopped for a few minutes in traffic. Confirmatory Factor Analysis of the Epworth Sleepiness Scale (ESS) with 1 factor and 2 factors among university students from the Brazilian Midwest, 2018 ( *n* = 400).

Figure 1 shows the CFA developed with a single factor and with 2 factors, identifying better adjustment measures in the factorial model with 2 factors (SRMR = 0.053, RMSEA = 0.095, CFI = 0.937, and TLI = 0.908, *p* = 0.001).

## DISCUSSION

The current study aimed to investigate the construct validity and internal consistency of the ESS for Brazilian medical students. Our findings showed appropriate psychometric properties, including an acceptable construct validity and internal consistency in this population group.

The ESS has presented itself as a solid scale, in psychometric terms, in several studies conducted around the world, with internal consistency and construct validity considered appropriate, 7 22 23 and demonstrating psychometric properties from acceptable to robust, 5 including high sensitivity and specificity in its original 4 as well as in translated versions. 5 22 23

Previous literature has shown that the ESS can be used for measuring the propensity to EDS in university populations of different nationalities and cultures. 6 7 22 23 However, the present study investigates the psychometric properties of the ESS in Brazilian university students, in whom adequate psychometric properties were observed in relation to construct validity and internal consistency of ESS.

In the present study, a good internal consistency (α = 0.75) was verified, corroborating the results of previous studies conducted with university students in Ethiopia (α = 0.75), 23 Peru (α = 0.85), Thailand (α = 0.78), 7 and India (α = 0.86). 22 A systematic review study on the psychometric properties of the ESS showed that Cronbach's α coefficients tend to range from 0.7 to 0.9, and lower values are generally found among non-clinical samples, such as students and community samples. 5

Although the ESS was originally developed and validated as a unifactorial scale, 1 24 in our study, the results of exploratory and confirmatory factor analyses revealed the 2-dimensionality of this scale among Brazilian university students, with the items related to the evaluation of drowsiness in a state of rest being grouped in factors 1 and 2, and drowsiness evaluated in activity. In this sense, previous studies have also demonstrated that the eight items of the ESS do not evaluate a one-dimensional construct, both in clinical populations 5 11 and non-clinical ones. 6 7 22 23

The identification of these two forms of drowsiness reflects the importance of the different types of situations in which the individual may present drowsiness, with the situations that allow EDS at rest being perfectly acceptable as a form of rest and momentary relaxation. However, sleepiness during activity brings great concerns, since this type is considered socially unacceptable because it is related to a higher probability of traffic accidents, occupational accidents, and directly interferes with the health of the individual. 25 26

With regard to this two-dimensionality of factors for the evaluation of sleepiness in resting and active situations, a study conducted with American and Austrian university students is in line with our results because they refer to this issue of passive and active sleepiness. 6 However, it is noteworthy that, unlike our study, the abovementioned study did not consider question 3 of the ESS (“sitting inactive in a public place setting, such as in a theater or a meeting”) as a factor that refers to situations of activity. The inclusion of question 3 in our study's characterization of drowsiness may be justified by the social phenomenon of fear of inadequacy in public settings, particularly concerning the inappropriateness of sleeping in public. The need to remain alert in social situations may induce a heightened state of vigilance that counteracts the sensation of fatigue, potentially increasing anxiety and, consequently, furthering vigilance, which can affect the perception of drowsiness. 27 Thus, even in situations in which the body is at rest, such as during a meeting or theater performance, the brain may remain in a heightened state of alertness in response to concerns about social behavior and the expectation of attentiveness.

The strengths of the present study include a relatively large sample size that was sufficient to perform exploratory and confirmatory factor analyses, including homogeneity between the two samples of university students explored. We highlight a standard administration of the instruments used in the study and methodological rigor in data collection. However, it is noteworthy that the present study was limited to evaluating the internal and construct validity of the ESS scale, not contemplating other forms of validity. Moreover, the modern era, marked by the pervasive use of smartphones and tablets, has substantially transformed sleep and wakefulness patterns, emphasizing the need to reassess the ESS. Integrating questions that address the impact of blue light exposure and electronic device use before bedtime could provide a more comprehensive assessment of sleepiness in contemporary life. Evidence suggests that interaction with digital technology not only affects sleep quality but also influences daytime sleepiness, 28 while digital content consumption may lead to visual overstimulation and circadian rhythm disruption. 29 Therefore, revising the scale to incorporate these modern influences is essential to understand how technological factors shape sleepiness in contemporary society.

In conclusion, the present study showed appropriate psychometric properties for the ESS in medical students, including an acceptable construct validity and internal consistency. Thus, the ESS may be considered as suitable to assess EDS in university students, especially medical students. A two-dimensional factor structure was identified, including factors for assessing drowsiness at rest and drowsiness in activity. However, further investigations on this factorial structure of the ESS need to be developed, since presenting EDS in activity is unusual and deserves to be explored.
